# Formulation, characterization and evaluation of mRNA-loaded dissolvable polymeric microneedles (RNApatch)

**DOI:** 10.1038/s41598-018-30290-3

**Published:** 2018-08-07

**Authors:** Kai Jun Koh, Yi Liu, Seng Han Lim, Xian Jun Loh, Lifeng Kang, Chee Yen Lim, Kyle K. L. Phua

**Affiliations:** 1 Department of Chemical and Biomolecular Engineering, Faculty of Engineering, National University of Singapore, 4 Engineering Drive 4, Singapore, 117585 Singapore; 2 Department of Pharmacy, Faculty of Science, National University of Singapore, Block S4A, Level 3, 18 Science Drive 4, Singapore, 117543 Singapore; 3 Institute of Materials Research and Engineering, 2 Fusionopolis Way, Innovis, #08-03, Singapore, 138634 Singapore; 4 Micropoint Technologies Pte. Ltd., 3 Soon Lee Street, #04-37 Pioneer Junction, Singapore, 627606 Singapore; 5 Present Address: Faculty of Pharmacy, University of Sydney, Pharmacy and Bank Building A15, Sydney, NSW 2006 Australia

## Abstract

In this paper, we report a proof of concept study on the fabrication, characterization and therapeutic evaluation of *in vitro* transcribed messenger RNA (mRNA) loaded in a dissolving microneedle patch (RNApatch). We show that low molecular weight polyvinylpyrrolidone (PVP) can directly be used without further purification for RNApatch fabrication with no detectable mRNA degradation. Physical and functional integrity of mRNA stored within the RNApatch are completely preserved for at least 2 weeks under ambient conditions. While the loading of mRNA into RNApatch is limited by the solubility of mRNA in concentrated PVP solution, mechanical strength of RNApatch is not compromised by the presence of mRNA. RNApatch can mediate *in vivo* transgene expression of mRNA encoding luciferase for up to 72 hours and transfection efficiency and kinetics mediated by RNApatch compares favorably to subcutaneous injection. Interestingly, mRNA transfection efficiency does not correlate with contact surface area but instead increases with deeper delivery depths. In an E.G7-OVA immunotherapy model, RNApatch induces slightly higher cellular and humoral immune responses compared to subcutaneous injection. In conclusion, RNApatch is a viable delivery platform for mRNA and represents an attractive option with significant translation potential for the delivery of mRNA therapeutics.

## Introduction

Since the first successful demonstration of mRNA based cancer vaccination by Nair and Boczkwoski in 1996^1^, the search for the optimal mRNA delivery system has begun. Encouraging results from preclinical and clinical studies^2–9^ have attracted an increasing number of gene delivery researchers into the field of mRNA therapeutics. Notably, the nanoparticle platform is the most frequently reported for mRNA delivery. The emphasis on the nanoparticle platform is in part due to application of established DNA and siRNA delivery systems for mRNA delivery. However it overlooked the microneedle delivery platform which is also a plausible delivery platform for naked mRNA for several reasons. Firstly, it was demonstrated by us^10^ and others^11–14^ that mRNA, when delivered subcutaneously in naked format, is surprisingly efficient in translating the encoded protein and in some instances more efficient than in nanoparticle format^10,13^. Hence, the robust performance of subcutaneously applied naked mRNA is a unique property that can be effectively exploited with the microneedle delivery platform. Secondly, an additional advantage of using naked mRNA is that it does not require the use of gene carriers, which complicate clinical translation due to the need to demonstrate safety and efficacy leading to high developmental cost and risk. Thirdly, skin targeted delivery of naked mRNA is expressed by both skin resident dendritic cells^15^ and non-immune cells^14^, making it ideal for inducing both cellular and humoral immune responses.

Cutaneous mRNA administration *in vivo* thus far has been mediated by the hypodermic needle: an effective delivery device yet associated with pain and anxiety. Consequently, it is often unpopular with patients^16,17^ and leads to poor patient compliance^18^. Recently, dissolving microneedles have garnered significant interest as a platform for cutaneous drug delivery. These devices are composed of micron-sized needle arrays formed by water-soluble polymeric or sugar excipients which contain the active drug. The excipients confer mechanical strength needed to disrupt the stratum corneum and allow needle entry into the viable layers of the skin. Upon penetration, they dissolve upon contact with interstitial fluids in the skin and release the encapsulated drug. A key advantage of delivering mRNA via the dissolvable microneedle platform is that it is delivered in solid form and hence obviates the need for handling mRNA in solution form, which not only avoids product damage caused by RNase contamination but also improves mRNA shelf life.

Despite the advantages of mRNA, existing work on gene therapy using dissolving microneedles focused mainly on DNA except for 2 reports on siRNA delivery^19,20^. This could be attributed to mRNA’s reputation as a relatively labile molecule and hence potentially challenging to be encapsulated stably in a microneedle platform. While mRNA can be relatively stable at room temperature^21^, it is easily susceptible to RNases which are highly stable and ubiquitously present on surfaces contaminated directly or indirectly through skin contact. In this work, we demonstrate for the first time that mRNA is sufficiently stable for manipulation and fabrication in dissolving microneedles. mRNA-loaded dissolving microneedles (RNApatch) are fabricated using the micromolding method followed by physical and functional characterization. We further evaluate the RNApatch therapeutically using a E.G7-OVA immunotherapy model to ascertain its ability to induce cellular and humoral immune responses. To the best of our knowledge, this is the first proof of concept study on use of dissolvable microneedles for mRNA delivery.

## Results and Discussion

### mRNA loaded into RNApatch remains intact and functional

Due to the labile nature of naked mRNA, its stability in concentrated polymer solution at room temperature for an extended period of time is a vital but missing piece of information. RNase contamination of the polymer is a major concern especially since RNases will be enriched at high polymer concentrations needed for RNApatch fabrication. Low molecular weight PVP is chosen as the excipient because it is a clinically approved polymer that possesses high mechanical strength, biocompatibility and water solubility^22^. In addition, PVP is a synthetically derived material, which reduces the likelihood of RNase contamination at source.

**Figure 1:**
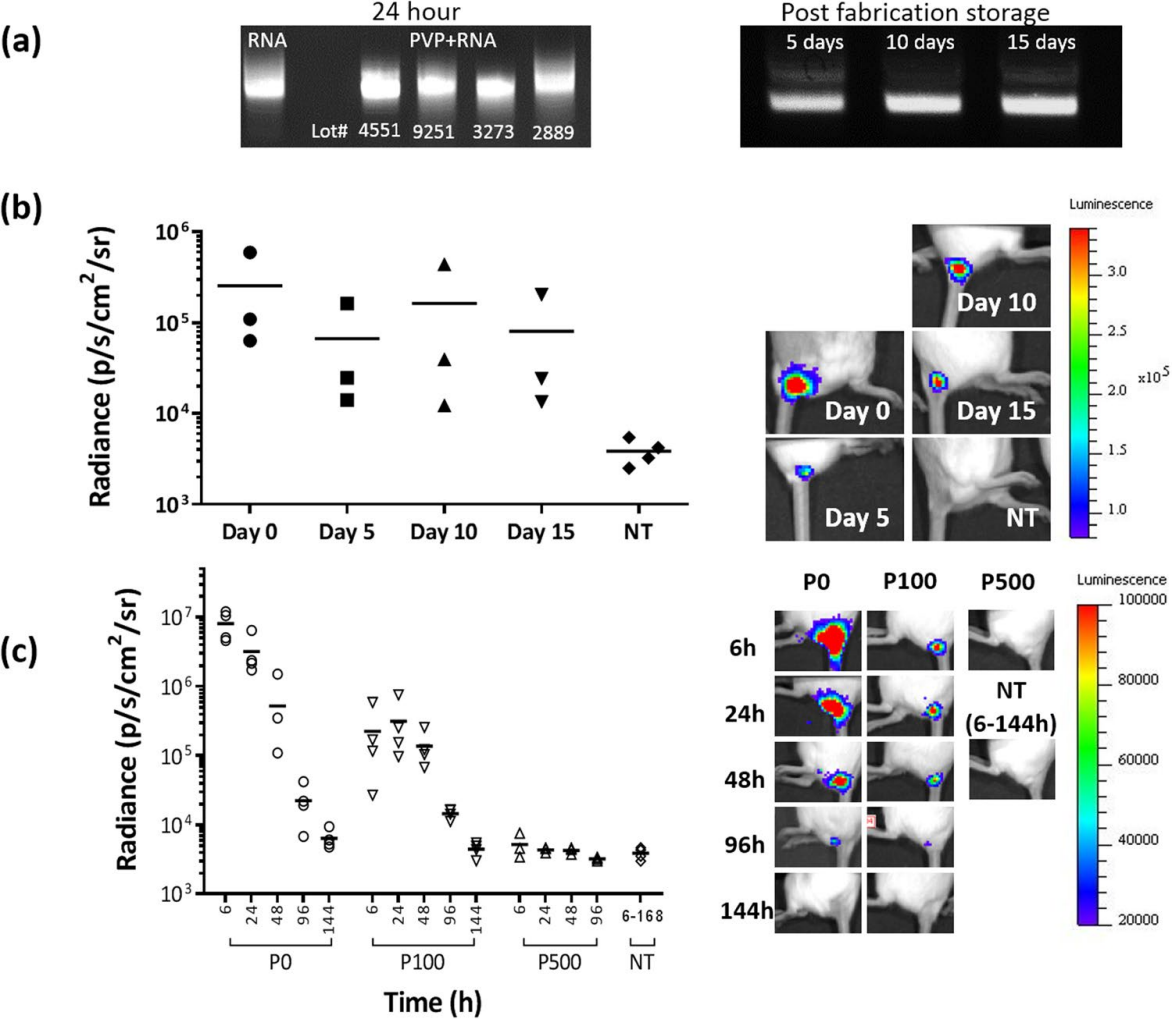
Effects of PVP on mRNA stability and function. (**a**) Gel electrophoresis of luciferase mRNA (mLuc) dissolved in PVP solution for 24 hours (left) and mLuc recovered from RNApatch 5, 10 and 15 days after fabrication was completed (right). (**b**) IcrTac mice were subcutaneously administered with 5 μg mLuc recovered from RNApatches 0, 5, 10 and 15 days after fabrication. Bioluminescence was assayed 6 hours post-injection. Representative IVIS images of correspondingly transfected mice are shown on the right panel. (**c**) IcrTac mice were subcutaneously administered with 6 μg of mLuc dissolved in various formulation over time. Bioluminescence was assayed at indication time points. P0: No polymer; P100: 100 g/L PVP; P500: 500 g/L PVP; NT: non-treated control. Representative IVIS images of correspondingly transfected mice are shown on the right panel.

Physical integrity of naked luciferase mRNA (mLuc) dissolved in PVP was evaluated using gel electrophoresis by comparing treated mLuc with untreated mLuc as a positive control. As shown in Fig. 1a, naked mLuc dissolved in PVP, taken directly from the bottle without further purification, and incubated for 24 h at room temperature remained intact. This was also observed in 4 different manufactured batches. In addition, physical integrity of mLuc recovered after being loaded into RNApatch from 5 to 15 days were also found to be well preserved. Moreover these mLuc remained completely functional *in vivo* as subcutaneously injected naked mLuc recovered from RNApatch (from 5 to 15 days) mediated comparable levels of luciferase expression (Fig. 1b).

Excessive amounts of PVP co-administered with mRNA may exert an inhibitory effect on mRNA transfection due to steric hindrance. To better understand the effect of PVP on transfection efficiency and kinetics of subcutaneously delivered mRNA, luciferase mRNA (mLuc) dissolved in PVP at different concentrations were injected subcutaneously into mice at the base of tail. P0 (0 g/L of PVP) and P100 (100 g/L of PVP), but not P500 (500 g/L of PVP) showed observable levels of bioluminescence at the transfection site up to at least 96 h post injection (Fig. 1c). The presence of PVP in P100 resulted in a reduction in initial transfection efficiency compared to P0 without significantly altering luciferase expression kinetics. On the other hand, an increase in concentration from 100 g/L (P100) to 500 g/L (P500) completely eliminated mLuc transfection. Since it has been reported that naked mRNA is taken up by cells via nucleic acid-specific receptor mediated endocytosis^14^, the drop in transfection efficiency can be attributed to steric crowding by PVP, which obscures mRNA from these receptors.

#### mRNA loading limited by solubility

**Figure 2:**
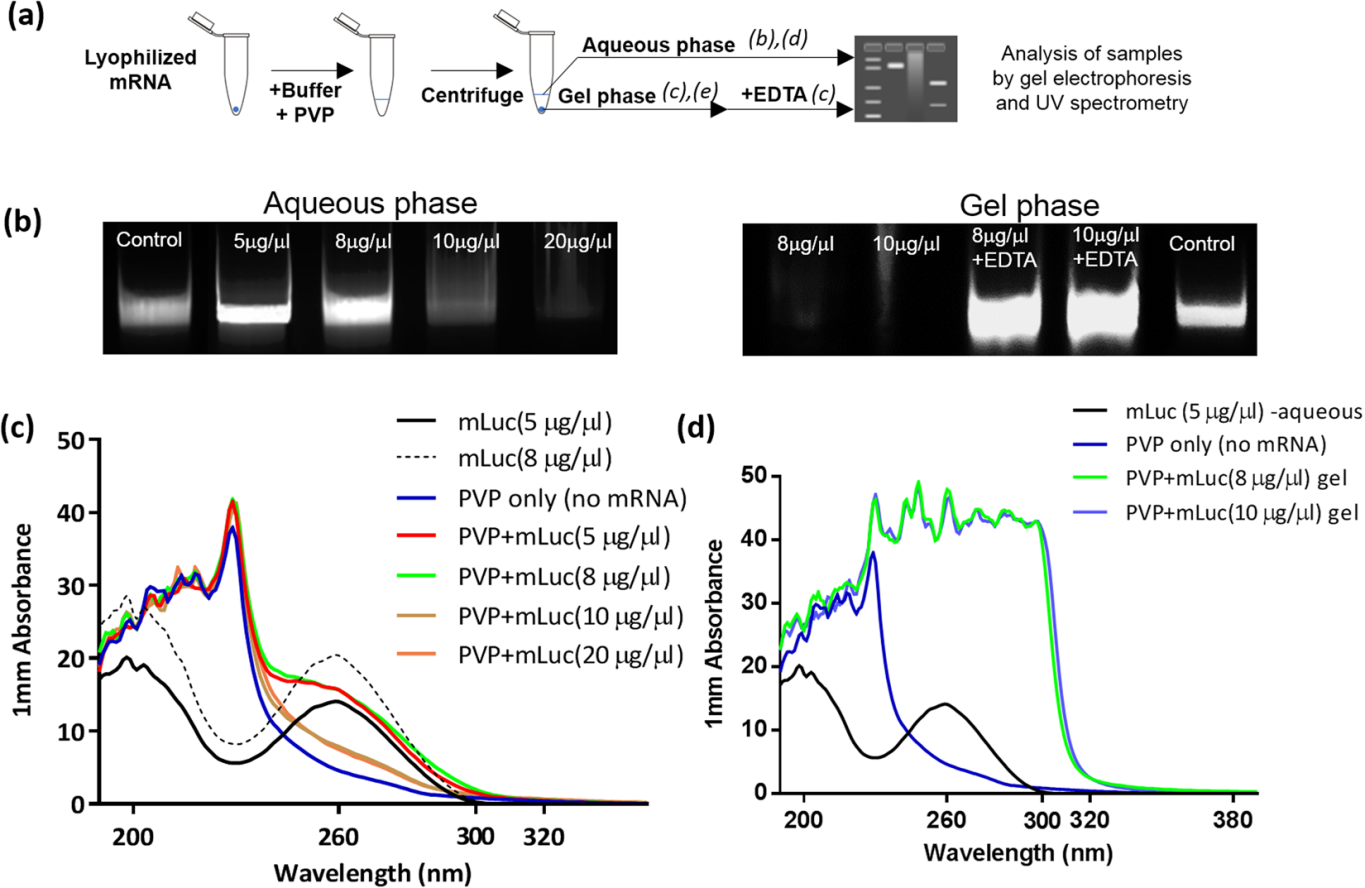
Solubility limit of mRNA in PVP solution. (**a**) Workflow for the evaluation of mRNA solubility limit in PVP solution. (**b**) Gel electrophoresis of mRNA dissolved in PVP at different concentration, aqueous phase (left) and gel electrophoresis of gel phase formed from high mRNA concentrations with and without EDTA (right). (**c**) UV absorbance of aqueous phases derived from mRNA-PVP samples. (**d**) UV absorbance of gel phases derived from high mRNA concentration mRNA-PVP samples.

The amount of mRNA that could be loaded in the PVP microneedles was limited by the solubility of mRNA in concentrated PVP solution. A transparent gel-like phase appeared within the otherwise aqueous PVP-mRNA mixture when final concentration of mRNA in the PVP exceeded 5 μg/μl. The gelation of mRNA/PVP was further analyzed, as shown in Fig. 2a, by separating gel and aqueous phases by centrifugation followed by gel electrophoresis. As shown in Fig. 2b (left panel), the amount of mRNA present in the aqueous phase as qualitatively indicated by the brightness of mRNA bands, did not increase when the final mRNA concentration was raised from 5 μg/μl to 8 μg/μl. Unexpectedly, the amount of mRNA present in the liquid phase declined at higher mRNA concentrations (10 μg/μl and 20 μg/μl) as indicated by the fading mRNA bands on the gel in Fig. 2b (left panel). Results from gel electrophoresis were further confirmed by UV spectroscopy. As shown in Fig. 2c, the aqueous phase UV absorbance (260 nm) of 5 μg/μl (red curve) and 8 μg/μl (green curve) samples were similar but significantly decreased at 10 μg/μl (brown curve) and 20 μg/μl (orange curve), respectively. On the other hand, as shown in Fig. 2d, gel phases of 8 μg/μl and 10 μg/μl samples showed UV absorbance that is significantly higher than either pure PVP or pure mRNA (Fig. 2b), indicative of gel formation. No gel phase could be recovered in the 5 μg/μl sample.

Gel phase from 8 μg/μl and 10 μg/μl samples were washed and EDTA was added as a dissociating agent to liberate the mRNA from the gel phase. After adding EDTA, gel phases were resolved as evidenced by well-defined mRNA bands recovered from EDTA treated samples (Fig. 2b, right panel). Hence the disappearance of mRNA from aqueous phase was due to the formation of a thermodynamically favored gel phase that sequestered almost all of the mRNA from the aqueous phase when mRNA concentration exceeded 5 μg/μl. Therefore dissolving mRNA in PVP above 5 μg/μl became counterproductive as it drove the formation of an mRNA-rich gel which could not flow into the microneedle mold. Consequently, all RNApatches were fabrication with mRNA/PVP mixtures containing 5 μg/μl.

**Figure 3:**
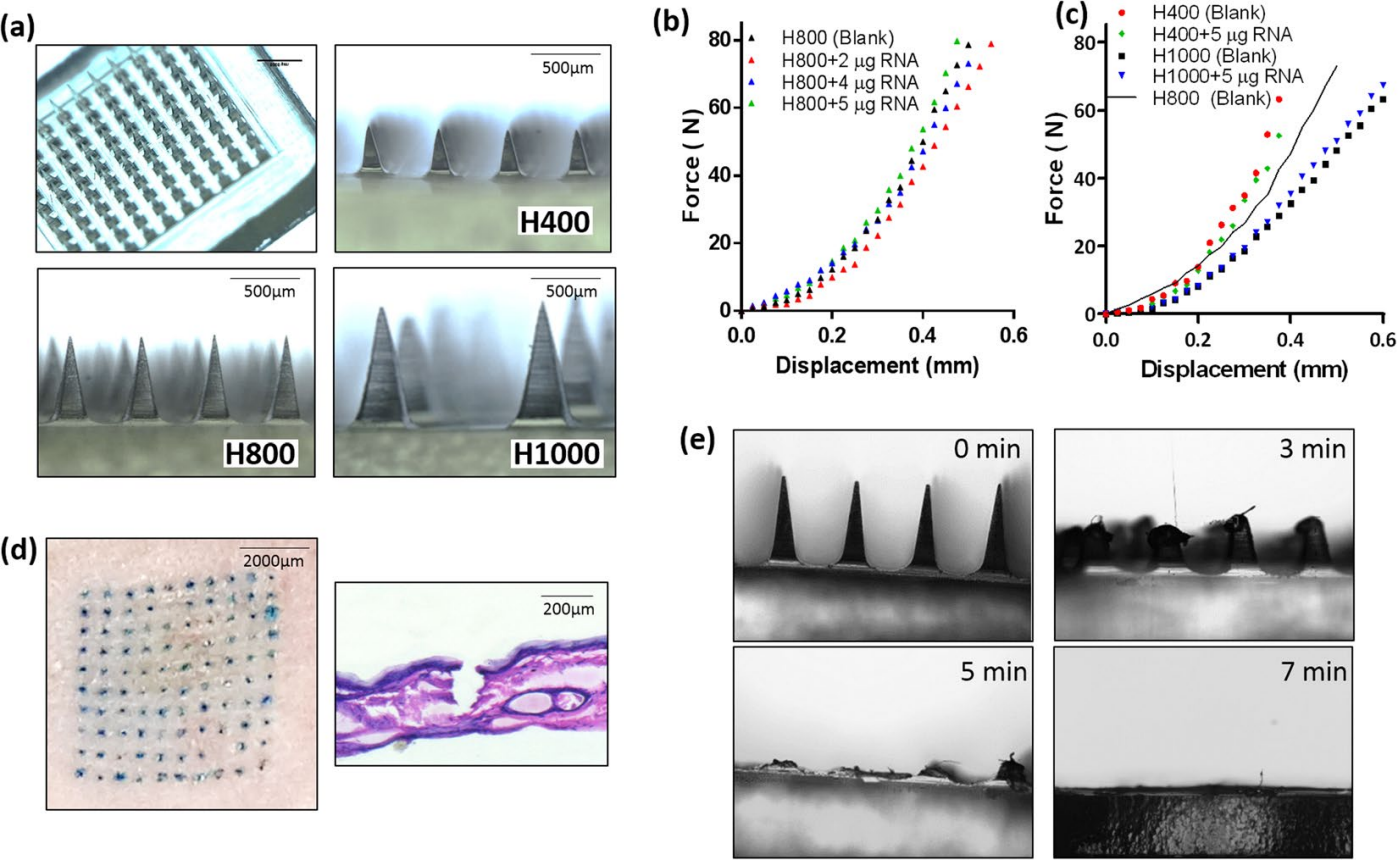
Physical characterization of RNApatch. (**a**) Bright field images of RNApatch, H400, H800, H1000. (**b**) Force-displacement graph of RNApatch H800 with varying mRNA-loading from 0 μg to 5 μg per patch. (**c**) Force-displacement graph of RNApatch H400 and H1000 with mRNA-loading of 0 and 5 μg per patch. (**d**) Mouse skin (left) after treatment with methylene blue loaded RNApatch H800. Hemotoxylin and eosin stained human cadaver skin (right) after administration by RNApatch H800. (**e**) Time course brightfield images of RNApatch H800 after being administered into skin of a live mouse and withdrawn at indicated time points.

RNApatches were fabricated using the micro-molding as described in the method section using PDMS molds with 3 different needle heights: 400 μm (H400), 800 μm (H800) and 1000 μm (H1000), respectively. Given that the volume of needle cavities in all mold types was about 1 μl, the maximum dose per patch was thus limited to 5 μg. We did not attempt to increase mRNA loading by reducing polymer concentration because of two reasons: Firstly, it was previously reported that expression of subcutaneously injected naked mRNA saturates at 5 μg^14^, hence we reasoned that this dose will be sufficient to demonstrate proof-of-concept. Secondly, given that H1000 was much weaker than H800 and H400 (Fig. 3b), a high polymer concentration was chosen to ensure sufficient mechanical strength of H1000 for fair comparison between the three types of RNApatches.

#### Fabrication of RNApatch

The resultant needle heights of the H400, H800 and H1000 were 341.2 ± 4.5 μm, 728.8 ± 7.4 μm and 916.67 ± 26.3 μm respectively (n = 5) (Table 1, Fig. 3a), which was a reduction between 9% to 15% in height compared to respective master structures. This reduction was due to volumetric contraction during drying^23^. For conciseness, RNApatches with the above needle heights would be referred as H400, H800 and H1000, respectively.

**Table 1:** RNApatch needle lengths, delivery depths and contact surface areas.

RNApatch	Nominal Needle length (μm)	Number of needles/patch	Actual needle length (Mean ± SD, μm)	Actual mRNA delivery depth (μm)	Contact surface area per patch (mm^2^)
H400	400	100	341.2 ± 4.5	200	16.0
H800	800	100	728.8 ± 7.4	400	32.0
H1000	1000	25	916.7 ± 26.3	500	17.8

Polymeric microneedles fabricated using pure PVP have been thoroughly demonstrated to possess sufficient mechanical strength for skin penetration^23,24^, but the impact of loading macromolecules such as mRNA in PVP microneedles has yet to determined. H800 was fabricated with variable mRNA loadings in RNApatch, and subsequently evaluated using compression testing where compression force-displacement data were recorded. As shown in Fig. 3b, mRNA loading of up to 5 μg per patch did not significantly affect the mechanical strength of PVP microneedles, as the same force deformed the microneedles with approximately the same displacement. Force-displacement profiles for H400 and H1000 were also obtained as shown in Fig. 3c. While mRNA loading had no effect on the mechanical strength of both H400 and H1000, it was observed that RNApatch with shorter needles were stronger than longer needles.

#### Insertion, dissolution and delivery depth of mRNA-loaded microneedles

H800 was found to insert into mouse skin very efficiently as shown by visible methylene blue dye deposited into mouse skin from H800 (loaded with methylene blue dye) with close to 100% efficiency (Fig. 3d, left). H800 was also sufficiently strong to be inserted into human cadaver skin (Fig. 3d, right). Dissolution rate of H800 was evaluated to determine the required duration of administration. H800 was administered onto the flank of mice for 3, 5 and 7 minutes, withdrawn from administration site and examined under a microscope. As shown in Fig. 3e, it was found that most of the needles dissolved after 5 minutes and completely disappeared after 7 minutes.

**Figure 4:**
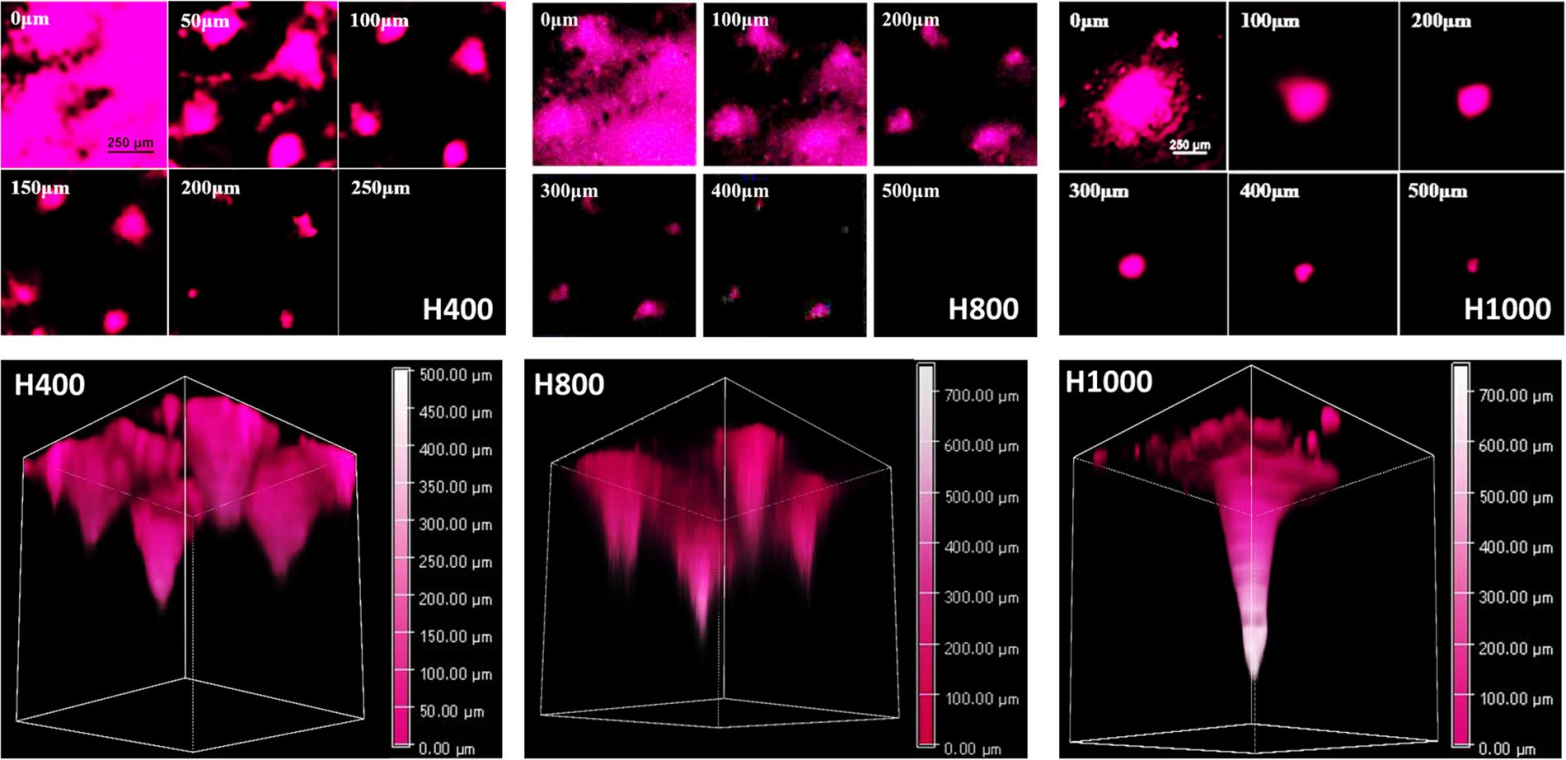
Determination of delivery depth of RNApatch H400, H800 and H1000 by confocal microscopy. Cross-sectional images (top panels) and 3D-reconstruction (bottom panels) images of mouse skin treated with indicated Cy5 labelled RNApatches.

RNApatches loaded with Cy5 labeled mRNA were administered onto freshly excised mouse skin and imaged at different depths using a confocal microscope. As shown in Fig. 4, H400, H800 and H1000 delivered Cy5 labeled mRNA to circa 200 μm, 400 μm and 500 μm, respectively, which confirmed that longer needles delivered mRNA to greater depths. 3D-reconstructions of Z-stack confocal images showed that the distribution of mRNA in the skin was geometrically consistent with the pyramidal shape of the needles, thereby confirming the observed delivery depths.

#### Transfection efficiency and variability increased with depth of delivery

**Figure 5:**
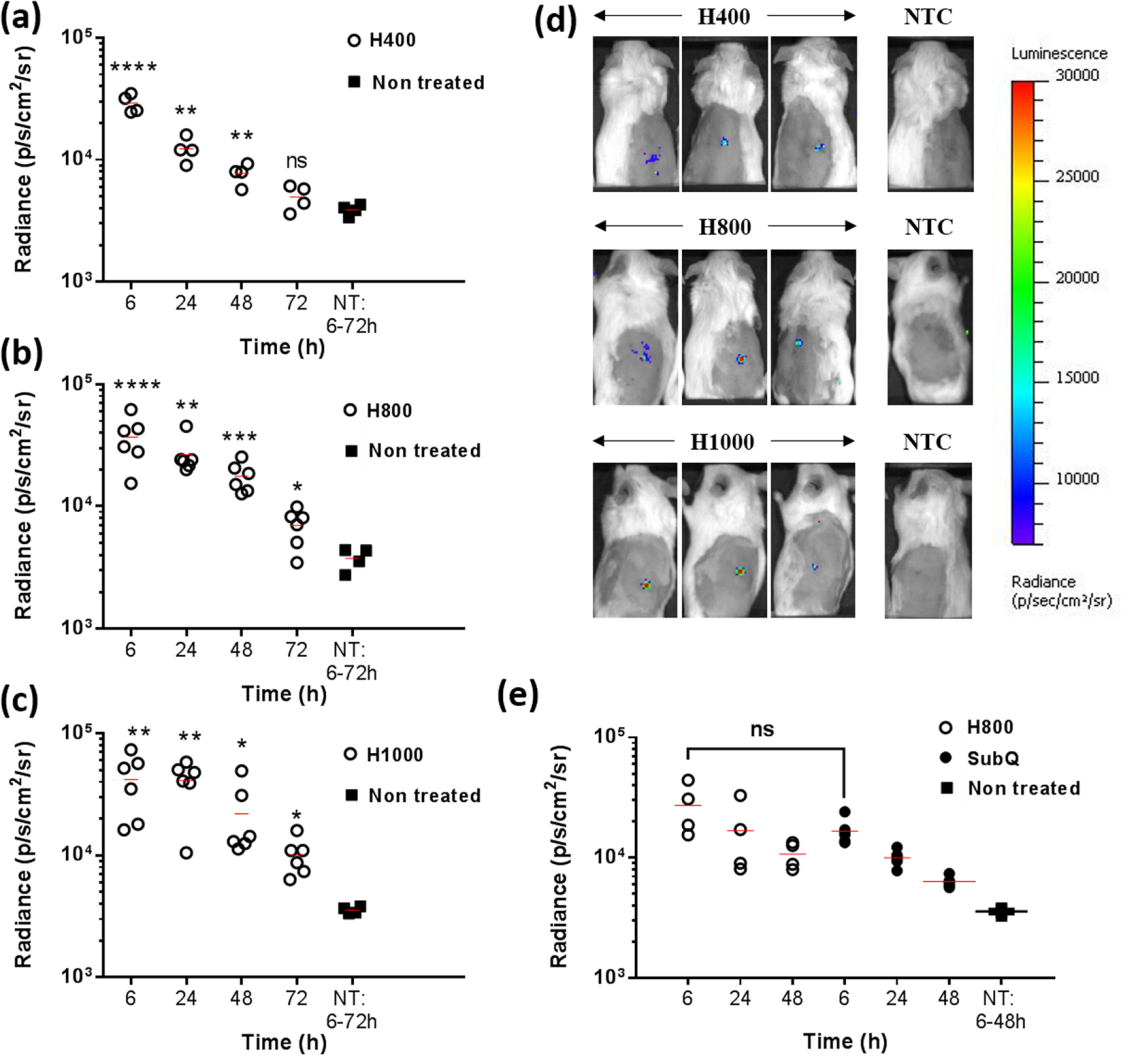
Transfection efficiency and expression kinetics of RNApatch. Mice were transfected with 5 μg luciferase mRNA via H400 (**a**), H800 (**b**) and H1000 (**c**) and luciferase expression was assayed at indicated time points. (**d**) Representative IVIS images showing luciferase expression of transfected mice 6 hours post transfection. (**e**) Luciferase expression in mice transfected with 5 μg luciferase mRNA delivered via H800 or subcutaneous injection. SubQ: subcutaneous injection; NT: non-treated control.

Mice were administered with H400, H800 and H1000 loaded with luciferase mRNA and the transfection efficiency as well as expression kinetics were monitored for up to 72 hours. As shown in Fig. 5a–d, luciferase expression was successfully achieved in all three types of RNApatch. It was noted that while RNApatch in this study was fabricated using highly concentrated PVP at 800 g/L, transfection was not adversely affected. This is because the total amount of PVP delivered by RNApatch (800 μg of PVP) was substantially lower than both P100 (4000 μg of PVP) and P500 (800 μg of PVP) as described in Fig. 1c, and would have been diluted after being dissolved by interstitial fluids.

H800 and H1000 both mediated higher transfection efficiencies and expression kinetics compared to H400, where luciferase expression reached background after 72 hours. While differences in transfection efficiency and expression kinetics between H800 and H1000 did not achieve statistical significance, mean radiance at all time points for H1000 were higher than H800 (Table 2). The standard deviation of transfection efficiency (both in terms of absolute value and percentage of mean) also increased with longer needles (Table 2). Since all three types of RNApatch delivered the same dose of 5 μg of luciferase mRNA, differences in transfection efficiency observed in Fig. 5 could be attributed to differences in delivery depths.

**Table 2:** Mean and standard deviation values of bioluminescence of RNApatch.

	6 hours		24 hours		48 hours	
	Mean (photons s^−1^ cm^−2^ sr^−1^)	SD (%)	Mean (photons s^−1^ cm^−2^ sr^−1^)	SD (%)	Mean (photons s^−1^ cm^−2^ sr^−1^)	SD (%)
H400	29200	5013 (17%)	12280	2862 (23%)	7740	1495.9 (19%)
H800	36825	15937 (43%)	26468	9432 (35%)	17632	4852 (28%)
H1000	41790	22697 (54%)	41023	16446 (40%)	21867	15233 (69%)

**Figure 6:**
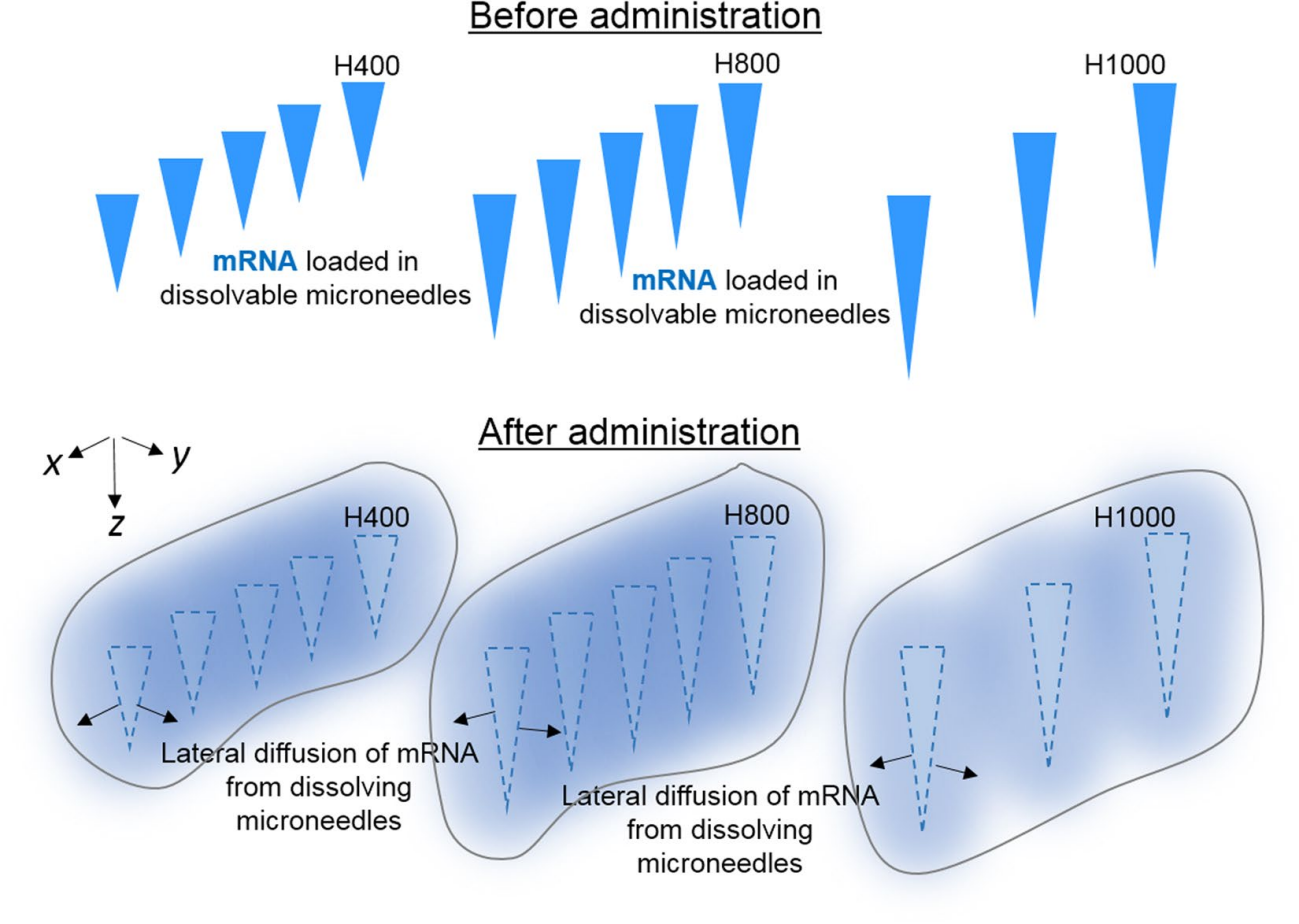
Illustration of lateral mRNA (blue) distribution from dissolving polymeric microneedles H400, H800 and H1000 that qualitatively accounted for high transfection efficiency of H1000 despite having a significantly lower contact surface area. The blue regions (within grey outlines) around the faded triangles denote mRNA distribution from dissolving polymeric microneedles (faded triangles) that correlated transfection efficiency data presented in Fig. 5.

Actual delivery depths and contact surface area (calculated based on actual delivery depths) for H400, H800 and H1000 were circa 200, 400 and 500 μm (Fig. 3) and 16 mm^2^, 32 mm^2^ and 17.8 mm^2^, respectively (Table 1). Our results (Fig. 5) showed that H400 with the lowest contact surface area, achieved the lowest average luciferase expression and kinetics. On the other hand H800 which had the same number of microneedles (100 per patch) but twice the contact surface area achieved a higher average luciferase expression and kinetics compared to H400. Since all RNApatches delivered the same dose of mRNA (i.e. 5 μg; volume of needle cavities for all RNApatches is 1 μl and loaded with 5 μg/μl of RNA/polymer), it could be reasoned that the higher transfection observed in H800 was attributed to a higher contact surface area as well as a greater delivery depth (Fig. 6). A higher contact surface area improved lateral distribution of mRNA within the skin (in the x-y directions) while deeper penetration increased mRNA distribution to additional layers of the skin (in the z direction). Both factors facilitated higher mRNA uptake by promoting greater cellular contact with mRNA.

Unexpectedly, the mean transfection efficiency mediated via H1000 (also delivering 5 μg of mRNA) was not lower than H800 despite having only 55% of H800’s contact surface area (Fig. 5, Tables 1 and 2). H1000 and H800 were identical in size (1 × 1 cm) but H1000 only had 25 needles per patch compared to 100 per patch in H800. Since naked mRNA transfection efficiency directly correlated with uptake^14^, we inferred that mRNA molecules could have diffused laterally resulting in efficient distribution of mRNA in the x-y direction regardless of needle pitch (i.e. distance between needles) as shown in Fig. 6. Furthermore, as increased transfection necessarily indicates a greater number of cells taking up the mRNA, we reasoned that the slightly higher transfection efficiency mediated by H1000 was also contributed by an increased penetration depth (Fig. 3, Table 1) and hence higher distribution in the z direction (Fig. 6). Taken together, we showed for the first time the impact of lateral diffusion of subcutaneously administered naked mRNA. This is a novel observation because it is widely accepted that naked mRNA, which has hitherto been delivered only via hypodermic needles, remains *in situ* after injection and requires to be taken up by migratory dendritic cells before showing up in the draining lymph nodes^12^.

H800 was selected for further study as it struck a balance between good transfection efficiency and moderate variations. A benchmarking study was performed to compare microneedle administration (H800) with subcutaneous injection (hypodermic needle). As shown in Fig. 5e, both delivery methods were effective and resulted in statistically significant levels of bioluminescence for 48 hours. Mice treated with H800 exhibited slightly higher average radiance at all time points from 6 to 48 hours post-transfection. However the difference was not statistically significant.

#### Anti-OVA immunotherapy using RNApatch H800 induces cellular and humoral anti-OVA immunity

**Figure 7:**
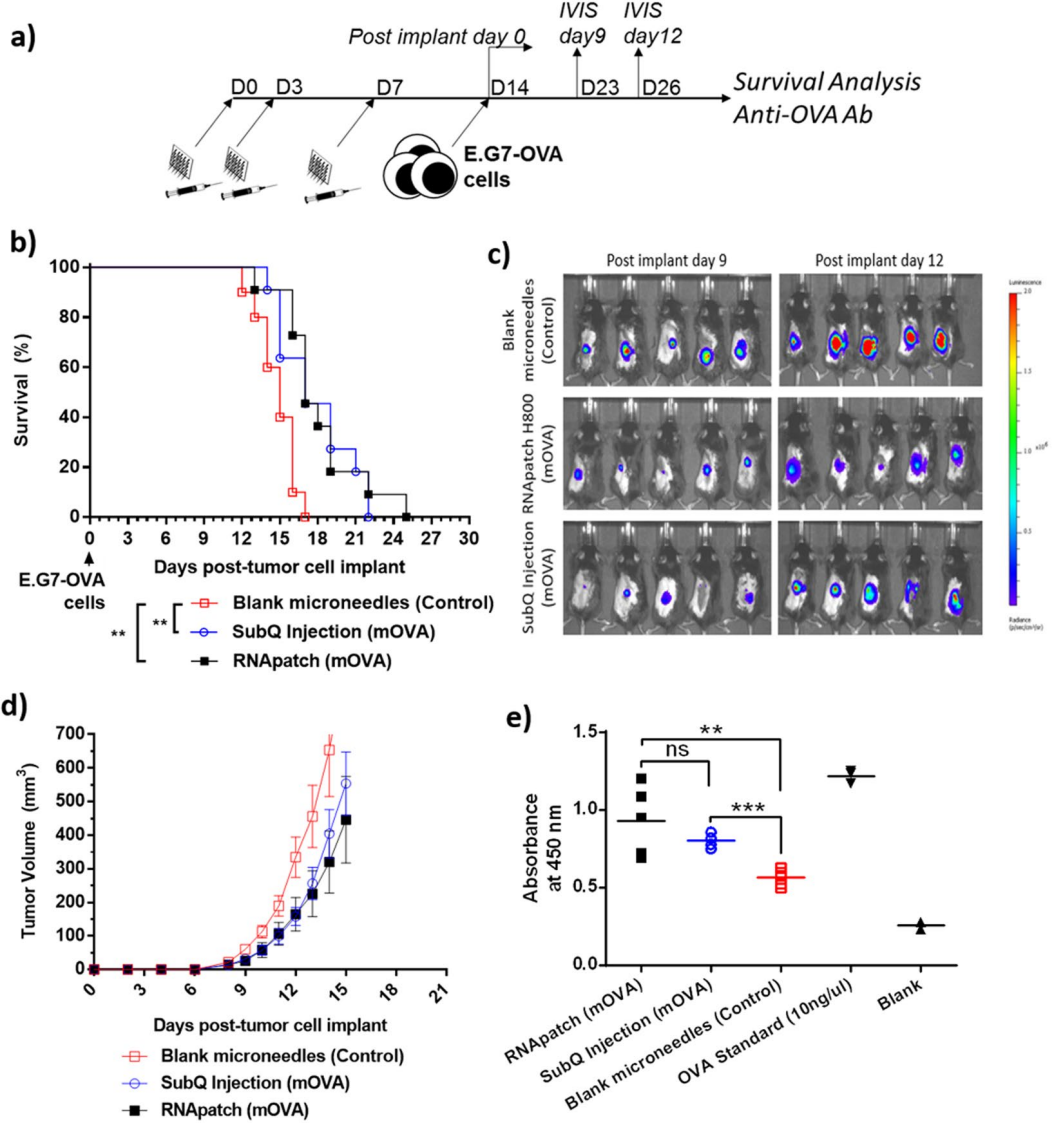
E.G7-OVA immunotherapy model to evaluate cellular and humoral immune responses from immunization with ovalbumin (OVA) mRNA delivered via RNApatch or subcutaneous injection. (**a**) Immunization and assay scheme. (**b**) Survival curve for mice transfected with OVA mRNA delivered via RNApatch or subcutaneous injection. Control mice were treated with blank microneedles. (**c**) Representative IVIS images of tumor bearing mice, visualizing tumor size through bioluminescence from luciferase expressing E.G7-OVA cells at indicated time points. (**d**) Average tumor volumes of tumor bearing mice from all three groups. (**e**) Serum anti-OVA antibodies analyzed using ELISA in all three groups.

E.G7-OVA immunotherapy model was used to evaluate the immunotherapeutic efficacy of RNApatch H800. E.G7-OVA cells are mouse thymoma EL4 cells that constitutively expresses chicken ovalbumin (OVA) and thus express OVA epitopes as a unique antigen. Cytotoxic T lymphocytes specific to OVA can be induced by immunization of mice with OVA mRNA delivered either via RNApatch H800 or subcutaneous injection. These OVA-specific cytotoxic T lymphocytes can specifically lyse E.G7-OVA cells leading to delayed tumor progression in vaccinated mice. The vaccination scheme was shown in Fig. 7a. Cell mediated immune response was measured via anti-tumor response against E.G7-OVA cells while humoral immune response was measured via anti-OVA antibody titers in serum. As shown in Fig. 7b, mice immunized with OVA mRNA via both H800 and subcutaneous injection showed statistically significant delayed tumor progression compared to blank microneedles controls. Delayed tumor progression in immunized mice was confirmed by IVIS images (Fig. 7c) taken on day 9 (post implant) when palpable tumors were detected in all control mice and day 12 (post implant) when the first tumor met the endpoint criteria. Delayed tumor progression was also confirmed by a slower increase in tumor volume from immunized mice (Fig. 7d). It can be further observed, based on bioluminescence of luciferase expressing E.G7-OVA cells (Fig. 7c), that delayed progression was more pronounced in mice immunized using H800 than in mice immunized using subcutaneous injection even though the survivor curves were similar. This was subsequently confirmed by a slightly lower average tumor volume observed in H800 treated mice after the final IVIS imaging on day 12 (post implant). Similarly, as shown in Fig. 7e, anti-OVA antibodies were detected in mice immunized with OVA mRNA via both H800 and subcutaneous injection. Although H800 treated mice had a higher average antibody titers compared to subcutaneously injected mice, the difference was not statistically significant due to large variations of anti-OVA antibody titers. Taken together, OVA mRNA delivered in naked format using H800 could induce both cellular and humoral immunity at least as effectively, if not better than using subcutaneously injection.

## Conclusion

In this study, we demonstrate for the first time the fabrication, characterization and evaluation of a basic mRNA delivery device based on dissolvable microneedles (RNApatch). We show that mRNA is sufficiently stable for manipulation and fabrication of the RNApatch, but mRNA loading is limited by solubility. The RNApatch is mechanically strong and is capable of mediating cutaneous delivery of naked mRNA. Transfection efficiency and kinetics improved with increased RNApatch needle lengths and are comparable with subcutaneous injection. Similarly, RNApatch induces equal prophylactic cellular and humoral immunity when compared to subcutaneous. In conclusion, the RNApatch is an attractive delivery device with significant translational potential for safe and efficient delivery for mRNA-based therapeutics.

## Experimental Section

### Ethics statement

All animal experiments were conducted in accordance with the guidelines stipulated during the Responsible Care and Use of Laboratory Animals (RCULA) training, as required by the Institutional Animal Care and Use Committee (IACUC) at the National University of Singapore (NUS). All studies reported in this work were also conducted under an approved protocol by NUS IACUC and NUS’s Institutional Review Board (IRB). The use of cadaver human skin for this study has been reviewed by the National University of Singapore Institutional Review Board (IRB in accordance to the NUS IRB-GUIDE-021 Guidelines on Research, using cadavers or cadaveric tissue specimens. The cadaveric tissue specimens were legally donated for research use after obtaining consent from the subjects or their next of kin.

### Materials and animals

RNase free water (RFW) was purchased from HyClone Laboratories. Tris-acetate-EDTA (TAE) buffer (10X) and agarose powder for gel electrophoresis was purchased from Vivantis. Ethidium bromide (EtBr) was purchased from Promega. Polyvinylpyrrolidone (PVP, 10 kDa) was purchased from Sigma-Aldrich. Polydimethylsiloxane (PDMS) micromolds for RNApatch fabrication were provided by Micropoint Technologies. For the *in vivo* luciferase assays, 18 weeks old female IcrTac mice (*In Vivos*) were used. For immunization experiments, 6–7 weeks old female C57BL/6 mice (*In Vivos*) were used. Isoflurane (Baxter) was used as a vaporized anesthetic when necessary. With the approval from the National University of Singapore Institutional Review Board, human dermatome skin was obtained from Science Care (Phoenix, AZ, USA).

### *In vitro* transcription and mRNA labelling

Capped and uncapped mRNA were synthesized using T7 High Yield RNA Synthesis Kit (E2040S, NEB) as per the manufacturer’s protocol and were reported previously^25^. Briefly, plasmid DNA containing a T7 promoter and poly-A tail (64 residues) was linearized and used as the template for IVT in the presence of Anti Reverse Cap Analog (ARCA) (NEB). mRNA was purified using RNEasy kit (Qiagen), the reaction yield was quantified using NanoDrop 2000 Spectrophotometer (Thermo Fischer Scientific) and full length mRNAs were visually confirmed by gel electrophoresis. Cy5-labelled mRNA were prepared using the Label IT® Nucleic Acid Labeling Kit (Mirus Bio) as per manufacturer’s protocol.

### Fabrication of RNApatch

RNApatches were fabricated by adapting the micro-molding technique that was previously reported^23,26,27^. PDMS molds with needle cavities of 1000 μm (H1000), 800 μm (H800) and 400 μm (H400) were provided by Micropoint Technologies. PVP solutions were prepared by dissolving 1.0 g of PVP (10 kDa) in 1 mL of Ringer’s Lactate. Known mass of mRNA were freeze-dried and reconstituted with a pre-determined volume of nuclease free water and mixed with PVP solution calculated to achieve a final mRNA and PVP concentrations of 5 μg/μL and 800 g/L, respectively. 30 μl of this mRNA/PVP solution was added onto the PDMS micromold and centrifuged (HETTICH, Universal 320) at 2800 g for 5 minutes to fill the needle cavities. Unused mRNA/PVP was recovered and the micromold was centrifuged for another 30 minutes. As microneedle cavities for H400, H800 and H1000 were 1 μL, the same amount of mRNA was loaded regardless of needle heights. Next, a blank solution consisting of 1.0 g PVP and 1.0 ml RFW was added and centrifuged for another 5 minutes to form the backing. The samples were kept at 50% relative humidity and room temperature for 2 days. The RNApatch was then removed from the mold and further dried with silica beads for 1 hour before use. Blank microneedle patches were fabricated using the same method without mRNA. Morphologies of the RNApatch were examined using a stereomicroscope (Nikon SMZ25) and the Nikon imaging software (NIS-Element Analysis D 4.20.00).

### mRNA stability

To ascertain the solution stability of mRNA in the presence of PVP, 1.0 μg of mRNA was incubated in PVP solution at 800 g/L at room temperature for 24 hours. To evaluate whether the fabrication process affected the mRNA integrity, mRNA was recovered from tips of RNApatches 5, 10, 15 days after fabrication was complete. mRNA for all samples including the control were drawn from the same tube to ensure consistency. All samples were analyzed by gel electrophoresis for 30 minutes on a 1.2% agarose gel containing ethidium bromide, and evaluated using G:Box Chemi XRQ (Syngene) imaging system. mRNA recovered from RNApatch after 0, 5, 10 and 15 days were also injected subcutaneously at base of tail and luciferase expression was assayed 6 h post administration.

### Excipient effect on mRNA transfection efficiency

To evaluate the effects of co-delivering PVP polymers during *in vivo* mRNA transfection, 15 mice were randomly split into 4 groups and each group was injected subcutaneously (base of tail) with 6 μg of mRNA dissolved in 40 μl of PVP at different concentrations. The positive control group (P0) was injected with 6 μg of mLuc in Ringer’s Lactate, without PVP; two experimental groups were injected with 6 μg mLuc in PVP dissolved in Ringer’s Lactate at 100 g/L (P100) and 500 g/L (P500). The negative control group (NT) was injected with Ringer’s Lactate without mRNA. Luciferase expression was monitored non-invasively at 6, 24, 47, 72, 96, 144 and 168 hours post-injection.

### *In vivo* luciferase assay

Luciferase expression was monitored using the IVIS Spectrum *in vivo* imaging system. Each mouse was injected intraperitoneally with 100 μl of luciferin (Gold Biotechnology, 28.5 mg/ml in PBS) at indicated time points before imaging. Non-transfected (NT) controls were always included on each platform and imaged at the same time to prevent false positives.

### Determination of loading and solubility

To determine the mass of mRNA loaded, mRNA loaded in a RNApatch was first separated by scraping the needles into an eppendorf tube and then re-dissolved with RNase-free water. The quantity of mRNA present was then determined using Nanodrop 2000 Spectrophotometer. A blank microneedle patch containing no mRNA was used as control to account for background absorbance caused by PVP.

To study the solubility limits of mRNA in the PVP formulation, known quantities of mRNA were lyophilized, re-constituted in nuclease free water and mixed with PVP (1000 g/L) in a 1:4 (v:v) to achieve desired concentrations of 5, 8, 10 and 20 μg/μl. After mixing, each sample was centrifuged using the Eppendorf 5424 Microcentrifuge at max speed for 1 minute. The supernatants were completely removed and transferred into new tubes for analysis. Gel pellets, if any, were washed by resuspending them with 100 μl nuclease free water, followed by centrifugation again at max speed for 1 minute. Supernatant were discarded and washed pellets, if any, were either directly used for analysis or further treated with 20 mM EDTA. Gel electrophoresis was employed to evaluate presence of mRNA in the supernatant, washed pellets, and EDTA treated washed pellets. The absorbance spectrum was characterized using Nanodrop 2000C Spectrophotometer using the UV-Vis mode.

### Mechanical properties of RNApatch

A digital force gauge (Algol, JSV-H1000) was used to characterize the mechanical strength of the RNApatch. The RNApatch was first placed on a flat surface with the needle tips facing upwards. A flat-head steel cylindrical probe was then moved downwards at a rate of (1.1 mm/s), perpendicularly to the flat surface, to apply an axial force to the needles. The digital force gauge then registered the amount of force required to achieve different displacements, up to a set limit of 80 N or 0.6 mm (0.4 mm in the case of H400), whichever was achieved first.

### Insertion and dissolution of RNApatch

The dissolution profile was determined by administering RNApatches on the flank of mice using a spring-loaded applicator from Micropoint Technologies. The hair on the flanks of mice were removed using an electric clipper followed by a depilatory cream (Veet, Reckitt Benckiser) one to two days before RNApatch administration. The RNApatch was administered onto bare skin of the anesthetized mice (isofluorane) and held in place for 3, 5, and 7 minutes. RNApatches were subsequently removed and residual microneedle projections were imaged. Penetration efficiency was investigated using H800 loaded with methylene blue (MB) fabricated as previously described except that MB (final concentration of 3 μg/μl) was loaded in lieu of mRNA. MB-loaded patch was administered onto a mouse for 7 minutes and the skin was wiped down to remove surface MB before visualization using the SMZ25 stereomicroscope.

Mechanical strength of the RNApatch was further characterized by evaluating its ability to penetrate stratum corneum of human cadaver skin. H800 was applied onto human cadaver skin using the spring-loaded applicator and quickly fixed with 10% neutral buffered formalin for 48 h. The sample was then transferred to 15% sucrose solution for 24 h and then frozen in an embedding matrix with liquid nitrogen immediately before sectioning. 10 μm cross-sections were obtained using cryostat (Lecia, CM3050S) and stained as per the standard hematoxylin and eosin (H&E) staining protocol. The samples were then imaged using the SMZ25 stereomicroscope.

### Determination of relative delivery depth and contact surface area

3 μg of Cy5-labelled luciferase mRNA was loaded into RNApatch (H400, H800, H1000, respectively) and administered onto freshly excised mouse skin mounted on 2 layers of parafilm. The skin samples were immediately mounted with Prolong Gold Antifade (Thermo Fischer Scientific), onto a glass slide and allowed to cure overnight under a cover slip. A confocal laser scanning microscope (Nikon, Eclipse Ti) was used to examine the sample under excitation at 633 nm to investigate the vertical distribution of the mRNA in the skin. Images were captured for the xy-plane at different z positions using the Z-stack function. The initial position (z = 0 μm) was defined as the skin surface based on bright field observation. 3D distribution of mRNA based on Cy-5 fluorescence was reconstructed from the xy-images using microscope software.

### *In vivo* reporter gene transfection efficiency and kinetics

16 mice were randomly divided into 3 groups for treatment with H400, H800 and H1000 each loaded with 5 μg of luciferase mRNA and were fabricated as described earlier. Each RNApatch was administered using the applicator and held in place for at least 7 minutes before removal. Luciferase expression was monitored at 6, 24, 48 and 72 hours after RNApatch administration. To compare the performance of RNApatch against subcutaneous injection, mice were administered with 5 μg of luciferase mRNA loaded in H800 RNApatch or dissolved in 60 μl of sodium acetate via hypodermic needles. Luicferase expression for both groups were monitored at 6, 24 and 48 hours post transfection.

### Tumor immunotherapy model

6 to 7-week old female C57BL/6 mice were immunized according to vaccination scheme shown in Fig. 6a. For each vaccination, mice were immunized with three RNApatches, each loaded with 3.4 μg of ovalbumin (OVA) mRNA and 1.6 μg of granulocyte-macrophage colony-stimulating factor (GM-CSF) mRNA. Alternatively, mice were immunized subcutaneously with 10 μg of OVA and 5μg GMCSF mRNA in 60μl of sodium acetate buffer. Control mice were treated with blank PVP microneedles loaded without mRNA. Seven days after the last immunization, 4 × 10^5^ luciferase expressing E.G7-OVA tumor cells (in 100 ml PBS) were injected subcutaneously into the left flanks of immunized mice. Mice were monitored for tumor onset and tumors were measured with vernier calipers. Tumor volume was calculated using the equation: Volume = ½ x Length/Width^2^, where length is the longer of the 2 orthogonal measurements. Mice with tumors greater or equal to 500 mm^3^ were considered to have met the end point criteria. At end point, serum was collected for ELISA before sacrifice. Anti-OVA IgG levels in serum were determined by ELISA using a mouse anti-OVA IgG Antibody assay kit (Chondrex, Inc) following manufacturer’s instructions.
